# Multidisciplinary approach to fused maxillary central incisors: a case report

**DOI:** 10.1186/1752-1947-8-398

**Published:** 2014-12-01

**Authors:** Gilberto Sammartino, Vincenzo Cerone, Roberta Gasparro, Francesco Riccitiello, Oreste Trosino

**Affiliations:** University of Naples Federico II, Naples, Italy

**Keywords:** Dental abnormalities, Endodontic treatment, Fused tooth, Gemination, Orthodontic treatment

## Abstract

**Introduction:**

The fusion of permanent teeth is a development anomaly of dental hard tissue. It may require a hard multidisciplinary approach with orthodontics, endodontics, surgery and prosthetics to solve aesthetic and functional problems.

**Case presentation:**

A 20-year-old Caucasian man presented to our Department to solve a dental anomaly of his upper central incisors. An oral investigation revealed the fusion of his maxillary central incisors and dyschromia of right central incisor. Vitality pulp tests were negative for lateral upper incisors and left central incisor. Radiographic examinations showed a fused tooth with two separate pulp chambers, two distinct roots and two separate root canals. There were also periapical lesions of central incisors and right lateral incisor, so he underwent endodontic treatment. Six months later, OPT examination revealed persistence of the periapical radiolucency, so endodontic surgery was performed, which included exeresis of the lesion, an apicoectomy and retrograde obturation with a reinforced zinc oxide-eugenol cement (SuperEBA) Complete healing of the lesion was obtained six months postoperatively. Fused teeth crowns were separated and orthodontic appliances were put in place. When correct teeth position was achieved (after nine months), the anterior teeth were prosthetically rehabilitated.

**Conclusion:**

Many treatment options have been proposed in the literature to solve cases of dental fusion. The best treatment plan depends on the nature of the anomaly, its location, the morphology of the pulp chamber and root canal system, the subgingival extent of the separation line, and the patient compliance. Following an analysis of radiographical and clinical data, it was possible to solve our patient’s dental anomaly with a multidisciplinary approach.

## Introduction

Fusion, concrescence and gemination are developmental anomalies of the dental hard tissue [1]. Tooth fusion is defined as the union between the dentin and/or enamel of two or more separate tooth germs [2]. If two adjacent teeth are connected by cement only, this is called concrescence [3]. The fusion may be partial or total, depending on the tooth development stage at the time of union. It may occur between teeth of the same dentition or between supernumerary teeth. In most cases, fused teeth show an anomalous size and shape of the crown, generally with separated roots, distinct pulp chambers and two independent endodontic systems.

Gemination, also called twinning, is a similar dental anomaly and it is defined as an attempt of the tooth bud to divide. Division is, in most cases, incomplete and results in a single root with only one root canal but two completely or incompletely separated crows [4].

Sometimes it is difficult to differentiate between fusion and germination, especially when fusion occurs between a normal and a supernumerary tooth [5]. To facilitate proper identification, teeth in the arch should be counted with the anomalous crown counted as one. A full dentition indicates gemination, while one tooth less than normal indicates fusion [6].

The etiology of these dental anomalies is unknown, but some causes have been suggested: the influence of pressure or physical forces producing close contact between two developing teeth, thus resulting in fusion [7]; close contact between two tooth germs that leads to necrosis of intervening tissue, allowing the enamel organ and the dental papilla to connect each other; and trauma and environmental factors such as thalidomide, embryopathy, fetal alcohol exposure or hypervitaminosis. In addition, some authors suggest that genetics may be an etiologic factor [8].

The prevalence of tooth fusion is estimated to be 0.5% to 2.5% in the primary dentition, whereas prevalence in the permanent dentition seems to be lower (0.1% to 1%) [9]. There is an overall lower incidence of double teeth in Caucasian than in Asian populations [10], and no differences in incidence between genders.

Fused teeth are mainly found in the anterior region, largely in the mandible, with incisors and canines the most frequently affected. In both primary and permanent dentitions fused or geminated teeth may cause functional, aesthetic, periodontal, orthodontic and caries problems. Several treatment methods have been described in the literature to solve these problems.

In this case report, we show how a multidisciplinary approach to fused maxillary central permanent incisors is necessary to achieve and re-establish proper function, shape and aesthetics of the affected teeth.

## Case presentation

A 20-year-old Caucasian man presented to our clinical Department for the correction of an excessively wide maxillary tooth and an anterior diastema that caused aesthetic and psychological problems. His medical history was noncontributory and there were no dental abnormalities among his family members.

An oral investigation revealed the dental anomaly and a missing tooth with regard to normal dentition. A diagnosis of fusion of two central incisors was made according to the definition of Braham [2].

**Figure 1:**
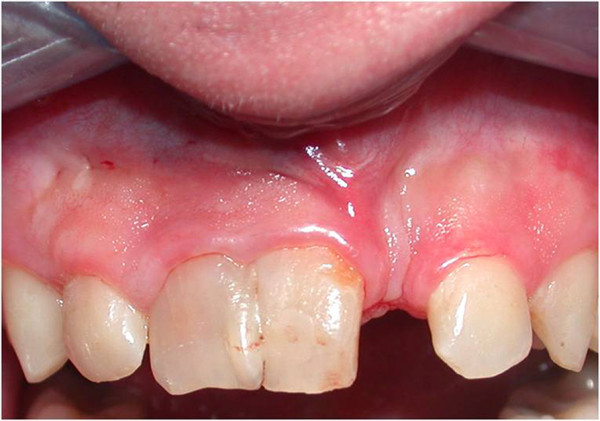
**Intraoral view.**

We also found a right central incisor dyschromia, a diastema between the fused teeth and lateral left incisor, and a dental misalignment. The fused teeth crown showed an evident palatal and buccal groove, extending 2mm subgingivally (Figure 1).

**Figure 2:**
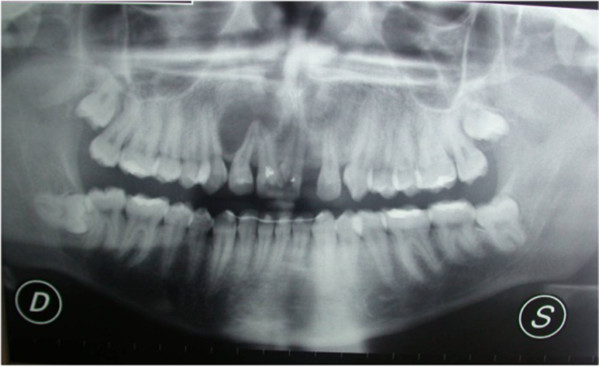
**Panoramic radiographic.**

Vitality pulp tests were negative for central incisors and right lateral incisor. A radiographic investigation showed a fused tooth with separate pulp chambers, two distinct roots and two separate root canals associated with periapical lesions of central incisors and right lateral incisor (Figure 2). A presumptive diagnosis of a radicular inflammatory cyst was made.

The treatment plan called for endodontic treatment, teeth separation followed by orthodontics, and prosthetic rehabilitation.

Because of the teeth necrosis and presence of a periapical lesion, conventional endodontic treatment was performed. His tooth was isolated with a rubber dam, an access cavity was prepared on the medial and distal part of his tooth and the pulp was extirpated. No communication was detected between the two pulp chambers using a curved probe. The root canals were cleaned and shaped, temporized with calcium hydroxide, and sealed. One week later, the endodontic treatment was completed.

**Figure 3:**
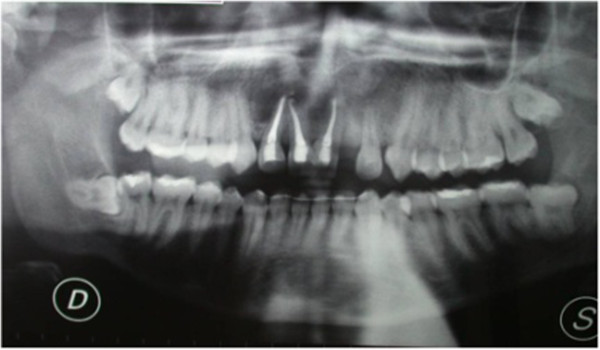
**Persistence of periapical radiolucency at six months control.**

**Figure 4:**
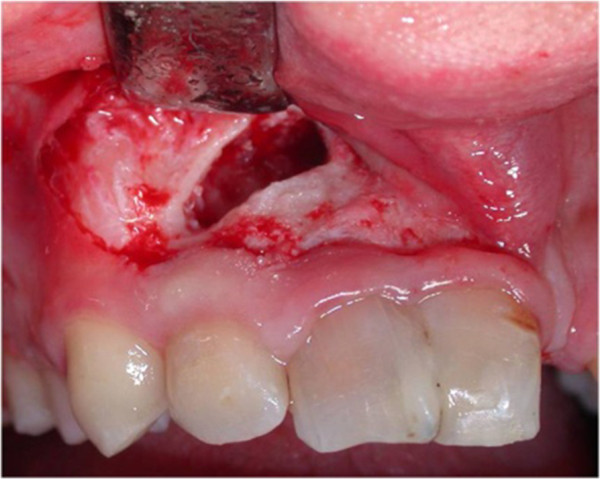
**Exeresis of lesions.**

After six months, a radiographic control revealed the persistence of periapical radiolucency (Figure 3). We then decided to perform endodontic surgery, which included exeresis of the lesions (Figure 4), apicoectomy, and retrograde obturation with a reinforced zinc oxide-eugenol cement (SuperEBA).

**Figure 5:**
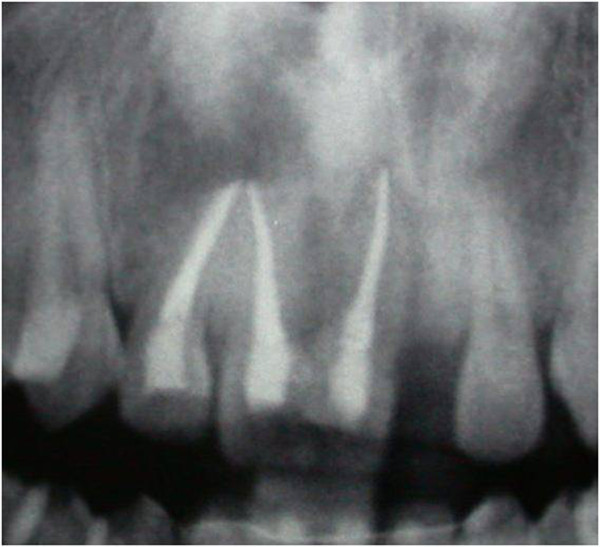
**Radiographic control at six months.**

**Figure 6:**
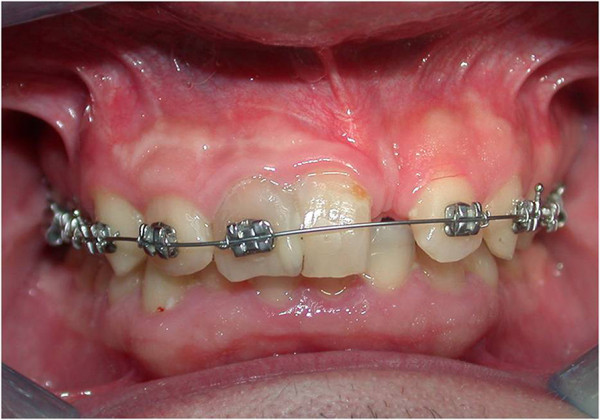
**Orthodontic appliances at initiation of treatment.**

**Figure 7:**
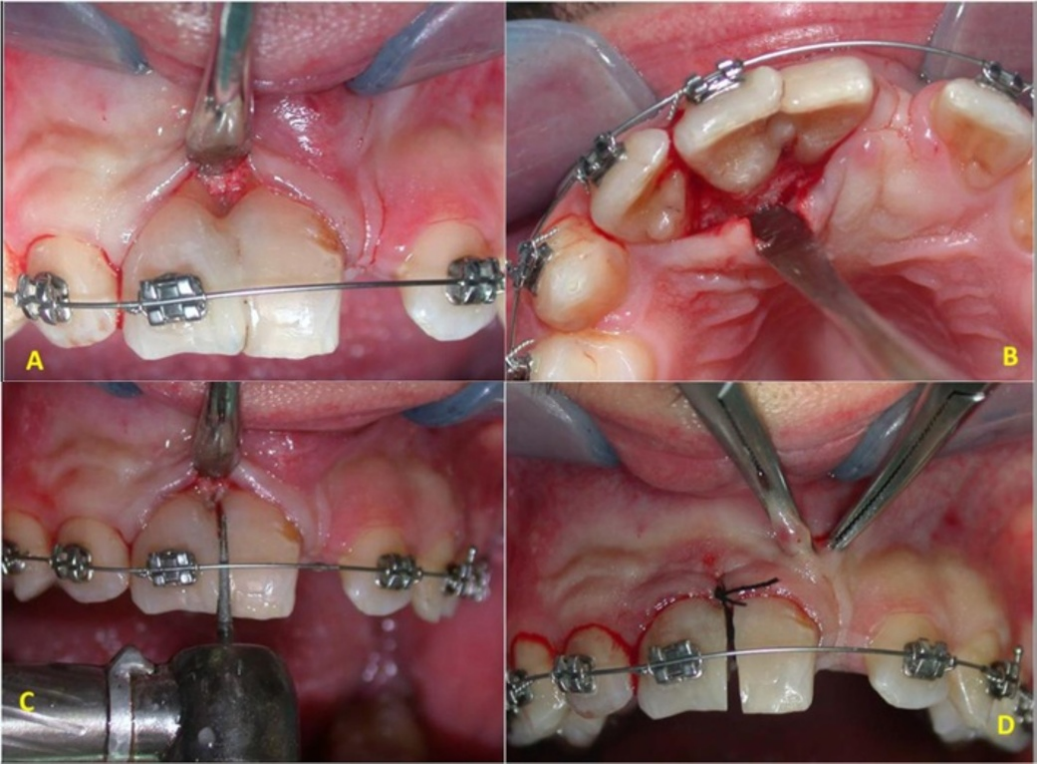
**Initial orthodontic treatment. (A,B)** Exposition of separation groove. **(C)** Separation of teeth. **(D)** Frenulectomy.

Complete healing of the lesion was obtained six months postoperatively (Figure 5) and orthodontic treatment was initiated after an evaluation of his molar class, overbite, overjet and so on. After one week, orthodontic appliances were put in place (Figure 6). At the same appointment, buccal and palatal flap were raised (Figure 7A,B) and the fused teeth crowns were separated along the buccal groove with a diamond bur (Figure 7C). Because of the presence of an anomalous labial frenulum, a frenulectomy was indicated (Figure 7D).

**Figure 8:**
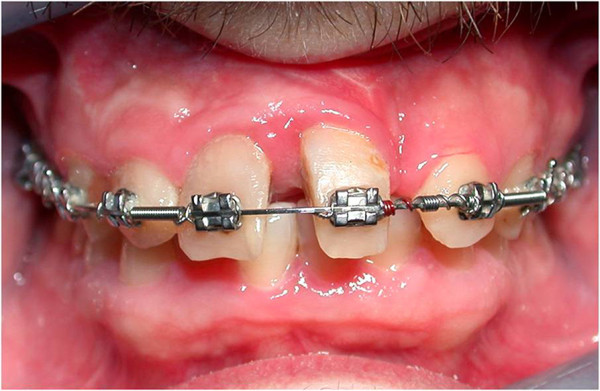
**Orthodontic treatment at nine months.**

**Figure 9:**
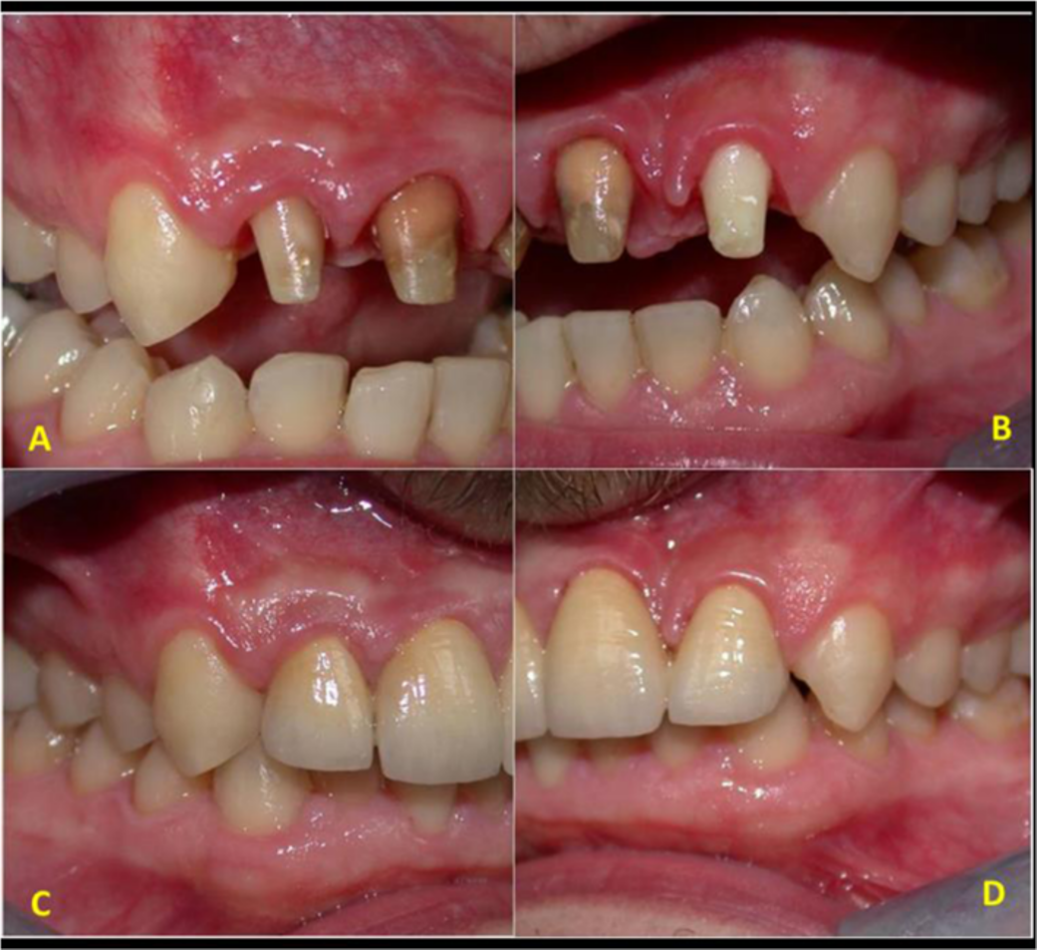
**Preparation and crowing of the anterior teeth. (A,B)** Preparation of teeth. **(C,D)** Provisional crowns.

Nine months later, the correct position of teeth was obtained (Figure 8) and the anterior teeth were prepared to receive crowns (Figure 9).

The provisional crowns were tested for two weeks before fabrication of the definitive prostheses to achieve the proper maturation of soft tissue. The definitive prostheses were made in ceramic material. The final aesthetic result was acceptable and our patient was satisfied.

**Figure 10:**
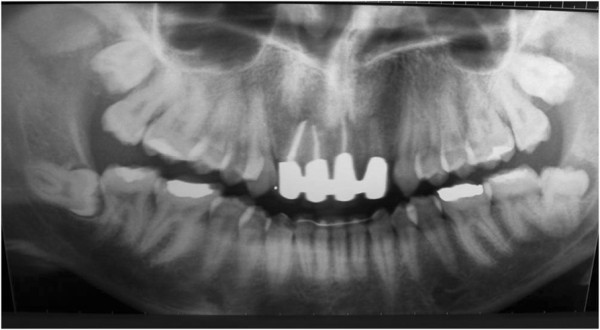
**One-year postoperative radiograph.**

A postoperative radiograph performed one year later showed no signs of periapical pathologies (Figure 10).

## Discussion

Fused teeth may lead to functional, orthodontic, endodontic and aesthetic problems that require multidisciplinary management [11]. Different treatment options have been described in the literature to overcome clinical problems caused by fused teeth [12, 13].

These treatment options depend on several factors, such as the type of abnormalities; the location of the connecting area; root development; patient age and compliance; and the pulp chamber and canal morphology.

Some reports recommend hemisection as the treatment of choice, although conservative treatment such as reconstruction of the margin crown and shape has been described [14, 15]. Hemisection and extraction of a part to treat fusion between teeth and supernumerary teeth is the common practice, followed by restoration of the remaining part and orthodontic treatment to realign teeth.

Another possible treatment is the extraction of the fused teeth and separation in the extraoral environment. This preserves the periodontal ligament and obtains a better hemisection line, and the tooth is replanted in the socket less than 5 minutes later.

Endodontic treatment should be performed when the pulp systems of both teeth are connected in a common pulp chamber or when communication exists between the two root canals. In our case, despite the presence of two separate pulp chambers and root canals, endodontic treatment was mandatory because of teeth necrosis of an unknown etiology.

Occasionally, leaving fused teeth untreated is proposed as an alternative treatment [11].

## Conclusions

Dental abnormalities can cause functional, orthodontic and endodontic problems, but problems are mostly aesthetic if located in the upper anterior region. They represent a challenge for clinicians because they require, in most cases, a multidisciplinary approach to achieve success and patient satisfaction. Clinicians must work together to choose the best possible treatment for the patient.

Endodontic therapy, as orthograde and/or surgical phase, is essential in these cases to reach a stability of healing that allows the other steps.

## Consent

Written informed consent was obtained from the patient for publication of this case report and accompanying images. A copy of the written consent is available for review by the Editor-in-Chief of this journal.
